# Neural Networks for Quantifying Laboratory Confocal Micro X-ray Fluorescence Measurements

**DOI:** 10.1021/acs.analchem.4c06545

**Published:** 2025-03-27

**Authors:** Frank Förste, Leona Bauer, Yannick Wagener, Felix Hilgerdenaar, Felix Möller, Birgit Kanngießer, Ioanna Mantouvalou

**Affiliations:** † Institute for Optics and Atomic Physics, Technical University of Berlin, Berlin 10623, Germany; ‡ Helmholtz-Zentrum Berlin for Materials and Energy, Berlin 14109, Germany

## Abstract

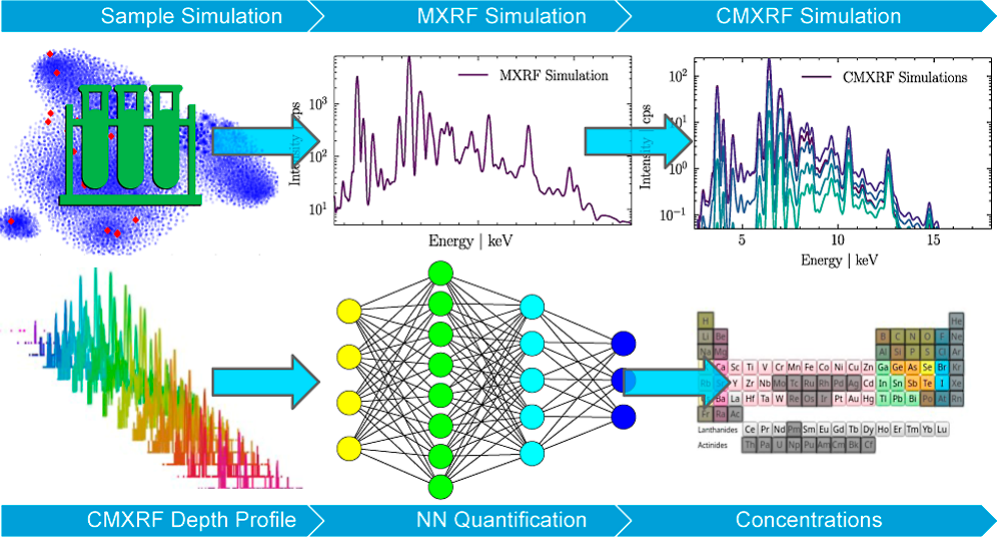

The quantification of confocal micro X-ray fluorescence spectroscopy (CMXRF) data obtained with polychromatic excitation in a laboratory setup is challenging. Complex dependencies, an elaborate setup calibration and nontrivial data evaluation makes it a time-consuming and intricate task. In this work we introduce the first application of a neural network for the quantification of homogeneous bulk samples, which significantly simplifies the evaluation and effectively eliminates the need for human input. The training of the neural network is performed on simulated data. For this, a simulation routine for CMXRF data of homogeneous bulk samples is introduced. The neural network is trained to simultaneously quantify the elemental concentrations of 53 elements, the density of the sample and the surface position directly from depth profiling measurements. As a result, the CMXRF evaluation is substantially simplified and the potential of the used neural network for feature extraction and prediction is demonstrated.

## Introduction

For various analytical research and industrial fields the knowledge of the elemental composition of a specimen in the micrometer regime is of major importance. A well established and reliable tool for this task is micro X-ray fluorescence spectroscopy (MXRF). It allows elemental imaging with 0D (point), 1D (line) and 2D (lateral) resolution of a sample qualitatively^1,2^ and quantitatively as e.g. elemental mass fractions.^3,4^ By adding a second optic in front of the detector MXRF can be performed in a confocal setup allowing 3D elemental imaging in the micrometer regime.^5,6^ Through the formation of a probing volume, confocal MXRF (CMXRF) thereby enables nondestructive 3D elemental analysis and was introduced and applied in multiple fields e.g. archaeometry, biology, cultural heritage and environmental studies.^7−10^ For depth resolved analysis, the sample is moved stepwise through the probing volume in the direction of the surface normal and spectra are measured as a function of depth position. For each position net peak intensities of the fluorescence peaks are extracted and yield so-called depth-profiles when plotted against the depth position.

For the quantification of elemental concentrations there are two common approaches for CMXRF, Monte Carlo and fundamental parameter (FP) based quantification, depending on the spectrometer setup.^11−14^ Following the procedure described in Mantouvalou et al.^12^ the analysis of depth-profiles is as follows: first the utilized spectrometer has to be fully characterized concerning the integral sensitivity and size of the probing volume. For this purpose, calibration measurements and procedures are applied. This is especially elaborate for laboratory setups using polychromatic sources, such as X-ray tubes, and has to be repeated prior to measurements in order to check and comply to setup changes. When the setup is fully characterized, the measured spectra of a depth-profile have to be deconvolved to extract the elemental fluorescence information. Elemental selection is performed manually. Depending on the composition of the sample, multiple fluorescence lines can overlap resulting in higher uncertainties in the deconvolution process. In the next step, a resource intensive and time-consuming iterative fitting has to be performed in order to retrieve the elemental concentrations. Well-matched starting parameters for the position of the sample surface, density and concentrations of the fluorescence elements are necessary. As not all elements can be detected with CMXRF, an assumption of this so-called dark matrix must also be given. For complex samples with many elements the fitting process can lead to long calculation times or even fitting failure. Therefore, a trained expert performs each of these steps.

In order to reduce the workload and complexity of CMXRF quantification, we introduce the first application of a machine learning-based quantification routine on bulk samples with a homogeneous elemental composition in 3D for CMXRF. While this is the first study of neural networks (NN) for CMXRF, studies in the related technique of XRF demonstrate the feasibility of NN for spectral analysis in the range of classification,^15^ identification^16^ and quantification of synchrotron^17^ and laboratory^18^ data.

Neural network training is solely performed on simulated CMXRF data. For this, a simulation routine for CMXRF depth-profiles of homogeneous bulk samples is introduced, which allows fast simulations of large data sets. The data was simulated for a wide range of calibrations of the used spectrometer, such as different geometries or alignments. The data therefore contains calibration information about the spectrometer as learning parameters for the NN. Further calibration of the utilized spectrometer is thereby redundant.

In the presented procedure, for successful quantification, a measured depth-profile is fed into the neural network, without the necessity for setup characterization, elemental selection, deconvolution or starting parameters. The NN returns simultaneously the elemental concentrations, the density and the surface position.

## Material and Methods

### Spectrometer

**Figure 1:**
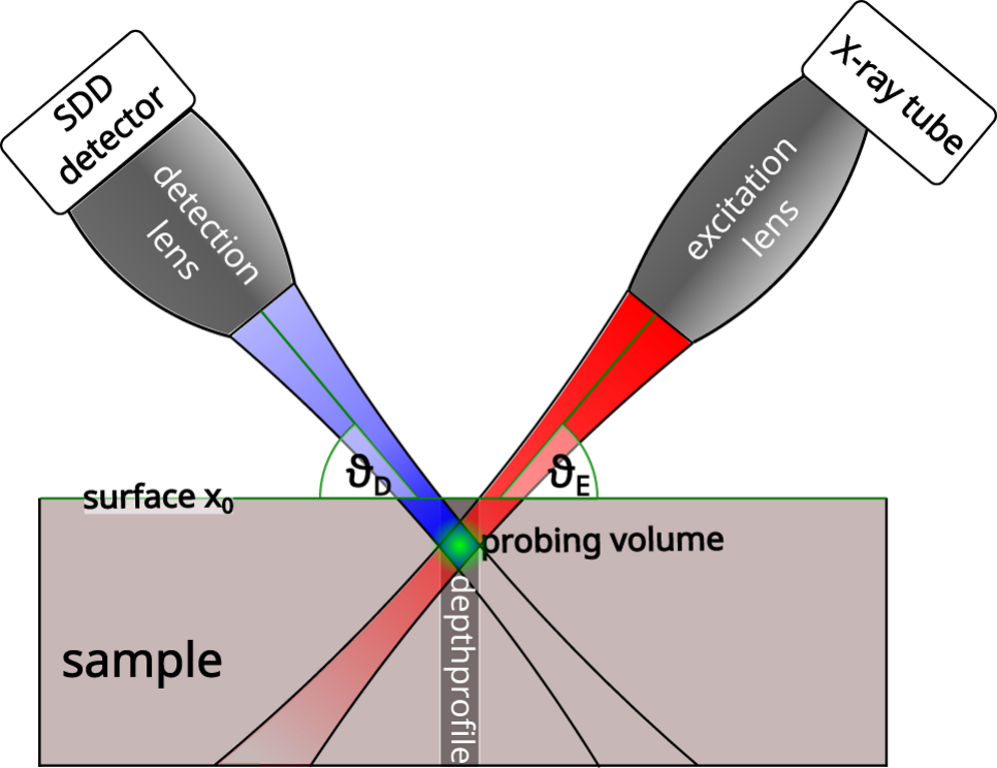
Displayed is a schematic sketch of a CMXRF setup. X-rays emitted from an X-ray source are focused on the sample by a polycapillary full lens. In front of the silicon drift detector a second polycapillary optic is mounted. The overlapping foci form a probing volume from which the fluorescence is detected. 3D fluorescence detection is achieved by moving the sample through the probing volume.

For all measurements in this work a modified commercial spectrometer (M4 Tornado Plus, Bruker Nano GmbH) was used. It is specifically adapted for CMXRF with a second polycapillary lens in front of a silicon drift detector (SDD).^19^ A schematic sketch of the setup is displayed in Figure 1. The spectrometer is equipped with a 50 W Rh-microfocus X-ray tube. The polycapillary lenses are mounted at the excitation and detection angle ϑ_E,D_ = 50° with respect to the sample surface plane. The tube parameters for all measurements were 50 kV and 1 mA and measurements were performed under ambient pressure. The sensitivity range of the spectrometer is from about 3 to 20 keV with a depth resolution (fwhm) of about 30 μm at the Cu Kα fluorescence energy. Depth-profiles are measured by moving the sample perpendicular to its surface and collecting spectra at specific depth steps. Depth-profiles were collected with 5 μm step size and different measurement times. The number of depth-profiles and measurement times are listed in the Table S1.

### Samples

Samples used in this work are either measured or synthetic samples. Measured samples are used for validation and testing, whereas the training is solely relying on synthetic samples.

### Measured Samples

The measured samples consist of reference materials with known elemental composition. They include multielement glass samples from Breitländer GmbH (BR A4, BR B2, BR C3, BR D3, BR E3, BR F3)^20^ (6) and pressed certified reference materials (CRM) of mostly biological and geological materials, as their relatively low density is well suited for CMXRF. The pressed CRMs are from the National Institute of Standards and Technology (NIST) (10), from the IAEA (5) and from the Joint Research Center of the European Commission: BCR (4). The samples are used to validate and analyze the performance of the trained NN. To test the extrapolation capabilities of the trained NN on samples not contained in the training data set the high-density CRMs Ferroetalon S25 and BAM M387, a steel and copper alloy, respectively, are also analyzed. Overall, 2152 depth-profiles of 27 CRMs were measured.

Additionally noncertified samples are measured. They consist of pressed pellets of the powdered BAM Umweltreferenzmaterial (URM)1^21^, cocoa bean material,^22^ cross-section samples from the plant parasite *Cuscuta reflexa*,^9^ a pressed pellet of powdered cellulose mixed with the multielement solution 042885.AE of Thermo Fisher Scientific Inc. and a cross-section of a bovine tooth. Overall, 143 depth-profiles on these 5 materials were measured.

The surface position *x*_0_ for all individual depth-profiles is determined by the software AbsCor,^23^ which performs FP quantification using an analytical fitting routine, and used as the ground truth for training.

All samples, except the alloy BAM M387 and steel Ferroetalon S25, are made up of more than 65%, most of them by even more than 90% of elements CMXRF is not sensitive to, since their fluorescence energies lie outside the sensitivity range of the used confocal setup. This so-called dark matrix heavily influences the scattering and absorption properties of the sample and thereby the measured spectra. Yet, single element concentrations of these elements cannot be quantified. In existing analysis approaches the composition of the dark matrix must therefore be predefined either by measuring these elements with different analytical methods or by using assumption about the matrix composition. The machine learning model presented here utilizes the absorption information on the dark matrix by analyzing the shape of the spectra of the depth-profile.

### Synthetic Samples

Collecting a sufficient amount of measurement data for NN training is not feasible in the field of quantitative CMXRF. On the one hand, a large number of reference materials would be required, on the other hand, measurement times would be significant since CMXRF measurements are time intensive. As a result, NN training must rely on simulation data. For successful training, a large diversity in the samples’ composition is necessary. For this, the elemental compositions of CRMs stored in the GeoReM database^24^ were utilized. For each element the occurrence rate in the database was calculated, and the respective elemental concentrations were approximated with a log–normal distribution. During sample simulation, the presence of each element in the sample was uniformly determined based on its occurrence rate. If present, its concentrations was drawn from the estimated log–normal distribution. This approach allows a minimized data set size since the simulated samples resemble real samples as close as possible. The elemental distributions and occurrence rates can be found in the Table S2.

Simulated elemental compositions were evaluated for their accuracy in representing measured samples, with a focus on the validation samples. For reducing dimensionality, t-distributed stochastic neighbor embedding (t-SNE)^25^ was applied to reduce the sample concentrations dimension from 98 to 2 dimensions. In the Figure S1 the reduced representation is visualized with the validation samples as red marker and the 22,080 synthetic samples used for training as blue marker. The parameters of the validation samples lie within the range of synthetic samples. Sample densities are uniformly drawn within the range of 0.5–3.5 g/cm^3^.

### Simulation

To date, no CMXRF simulation routine, software or algorithm has been published to the best of our knowledge. Therefore, we introduce a simulation routine here. As a first step, MXRF spectra are simulated using the software XMI-MSIM.^26^ Parameters for these simulations were adapted to MXRF measurements on the Breitländer glass references with the used spectrometer.

To convert an MXRF spectrum into a CMXRF depth-profile, a procedure based on the fundamental parameter equation described in Förste et al.^23^ was developed. The transformation from an MXRF spectrum to a CMXRF depth-profile can be conducted by calculating the following equation for each depth *x*_*n*_ relative to the surface position *x*_0_
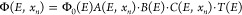
1with Φ(*E*,*x*_*n*_) fluorescence intensity at energy *E* and position *x*_*n*_ and Φ_0_(*E*) the MXRF spectrum. A detailed description deriving eq 1 is given in the Supporting Information. The transformation can thus be performed using the 6 setup parameters *T*_A_, *T*_B_, *T*_M_, σ^MAX^, σ^exp^ and σ^OFF^ and the surface position *x*_0_. From a simulated MXRF spectrum 40 CMXRF spectra with 5 μm depth distance are calculated. This set of spectra represents one CMXRF depth-profile.

The 6 spectrometer parameters define the setup used. To include changes in alignment and source fluctuations, these parameters were set variable during the conversion. The ranges used are listed in the Table S3. With the assumption of constant excitation conditions and that the MXRF setup does not change significantly, a time-consuming CMXRF calibration of the setup is thereby redundant.

**Figure 2:**
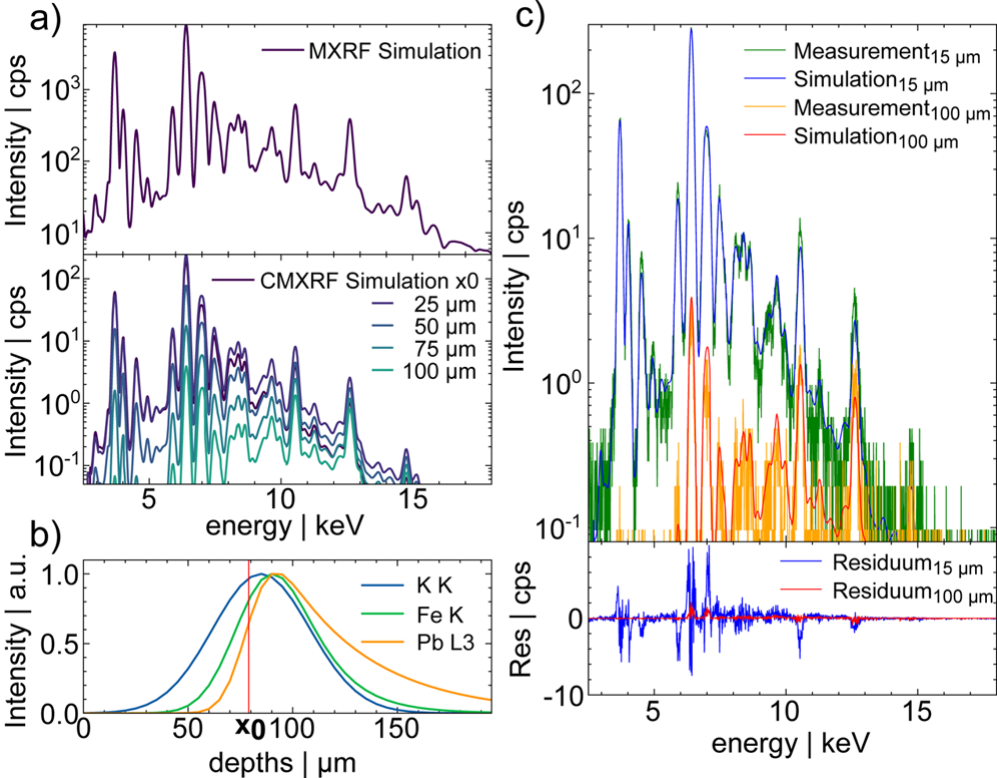
In all plots data of BR B2 are displayed. In (a), in the upper graph the simulated MXRF spectrum and below transformed CMXRF spectra from different depths relative from the sample surface *x*_0_ from purple to cyan are plotted. Due to absorption the intensity decreases, and the center of the spectrum shifts to higher energies with larger depth. In (b), normalized depth-profiles for fluorescence at different energies are displayed. In (c), measured and simulated CMXRF spectra at 2 different depths relative from the sample surface *x*_0_ are shown. In the lower plot their absolute deviation is shown.

The different steps of the transformation are displayed schematically for the multielement glass BR B2 in Figure 2. First, the simulated MXRF spectrum is displayed in the upper plot of (a). In the lower plot, transformed CMXRF spectra at different depths, from purple (surface) to cyan (approximately 100 μm below surface), are displayed. Note the decrease of intensity at higher and lower energies due to the transmission of the second optic. With rising depth, the intensity decreases, and the center of the spectra shifts to higher energy due to absorption. In (b) normalized depth-profiles for three different fluorescence energies are displayed. The surface position is shown as a vertical red bar. The profiles show the characteristic shape with a steep rise to a maximum after the surface followed by a decrease with varying steepness depending on the density of the sample and energy of the fluorescence. The simulated depth-profiles are smooth, which is not always true for measured samples, which display, depending on the inhomogeneity and measurement time, fluctuations. In (c) the difference between measured and simulated depth-profiles for two spectra at different depths are displayed along with their absolute deviation in the lower plot. They are in good agreement, with deviations below 10% in the fluorescence lines. The background is slightly overestimated in the simulation which was also observed by Rakotondrajoa and Radtke.^17^ This simulation-to-reality gap might lead to higher deviations in trace elements with low fluorescence intensity.

The approach to first simulate a MXRF spectrum and then convert it to CMXRF was chosen for three reasons: first, it allows a higher rate of depth-profile simulations since only one MXRF spectrum per sample needs to be simulated. Depending on the sample complexity the MXRF simulation takes about 15 s. Second, it is setup independent in that case that the simulated MXRF spectrum can afterward be transformed to any spectrometer configuration with the same excitation. And third, the transformation is fast, with about 0.4 s per depth-profile, improving scalability. Overall, 22,080 MXRF spectra and their depth-profiles have been simulated, resulting in approximately 95 h to create the training data set on a standard PC.

## Machine Learning Algorithm

Established CMXRF analysis routines were outlined in the introduction. Primary features extracted from CMXRF data include the fluorescence line energy position and intensity for each peak in the spectrum. Another important aspect is the behavior of detected intensity profiles with respect to depth. These profiles reveal the sample surface position and the energy-dependent absorption, which is dependent on the sample’s density and overall composition.

In this work, these tasks are transferred to a deep neural network. The data consists of the two-dimensional, energy-dependent intensity as a function of depth. A fluorescence peak at a specific energy confirms the presence of a particular element. Convolutional neural networks (CNNs) are suited for this task due to their sensitivity to local feature detection.^27,28^ Additionally, the intensity depth-profile contains information about surface position and absorption within the sample, which can also be effectively handled and analyzed by a CNN. A CNN utilized on both these tasks should also be capable of separating overlapping fluorescence lines. Due to the energy dependent absorption with depth the fluorescence intensities of overlapping lines changes differently, modifying the ratio of the fluorescence intensity creating a feature extractable by CNNs.

Final steps involve predicting concentration values by interpreting the depth-profiles. Multilayer perceptrons (MLPs) are commonly employed for similar tasks.^29^ The complete structure of the machine learning model for quantification consists of a CNN for feature extraction, followed by a fully connected MLP for prediction. NN features are listed in Table S4.

Model training was conducted exclusively on simulated CMXRF depth-profiles. To rely solely on simulated data is feasible as shown e.g. by Minor et al.^30^ As the loss function the mean squared error (MSE) is used. The learning rate, as an important parameter for neural network training, was set to 10^–4^. The output from the final layer produces a 55-entry vector, predicting concentrations for 53 elements—ranging from K to Nb, Cd to W, and Pt to Bi, excluding the noble gases Kr and Xe—along with density ρ and surface position *x*_0_. Element selection is based on the sensitivity of the setup, see the spectrometer section. Also, fluorescence energies below 3 keV are rapidly absorbed in a sample which results in shallow detection depths unsuitable for depth resolved measurements. During the training, Poisson noise is introduced on the depth-profiles to comply with noise in the measured data.

The training took approximately 4 h on a GeForce RTX 3060 GPU. The model has a size of 81.5 MB. It was implemented using PyTorch^31^ and Python 3.9^32^.

## Results

**Figure 3:**
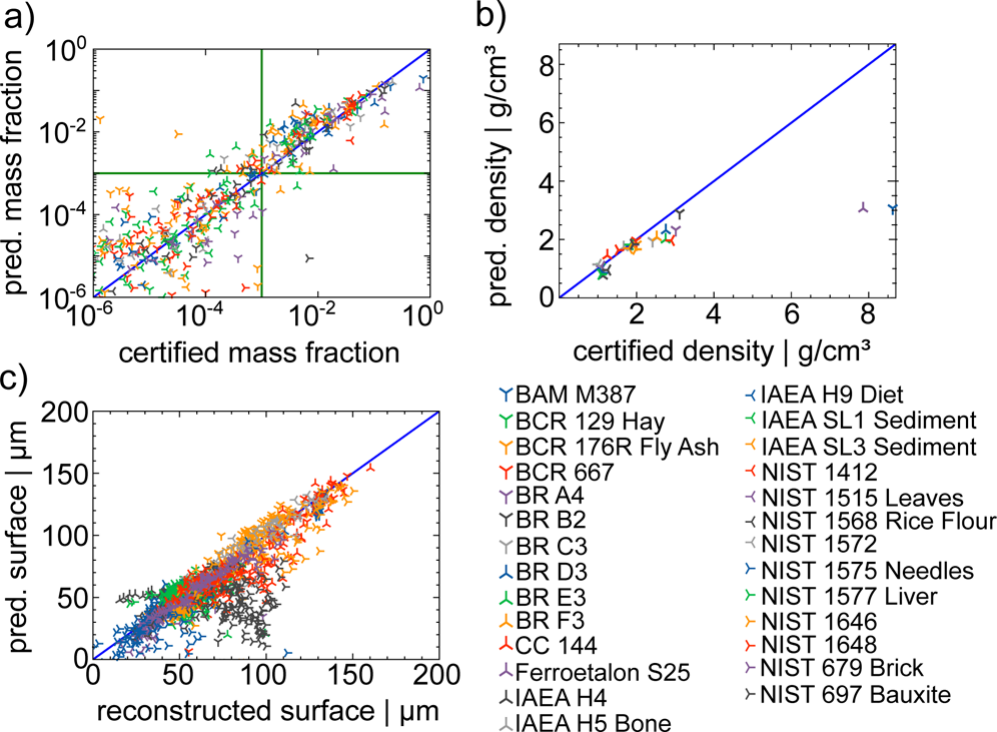
Predicted values as a function of the certified for (a) concentrations, (b) density and (c) surface position *x*_0_ of all CRMs and all elements represented are plotted with different symbols. The blue line represents perfect agreement. The green lines mark concentrations of 0.1%.

To analyze the trained model, a prediction on all measured reference validation data was performed. The prediction with 12 ms per depth-profile is fast, approximately 100,000 times faster than the FP approach with about 20 s per element or 15 min for the same number of elements. For each depth-profile of every CRM as listed in the Table S1 the concentration of elements, density and surface position *x*_0_ were predicted by the trained model. Mean elemental concentration and density values of the predictions on each measured depth-profile of each CRM are set as the quantitative results. A first qualitative analysis of the network performance can be conducted by analyzing the plots in Figure 3. In these plots, the predicted values are plotted as a function of their certified values. Different markers represent different CRMs. The blue line represents perfect agreement and model predictions should align along this line. Prediction accuracy is defined by the relative difference between the certified values and the prediction for concentrations and density, for the surface position absolute deviations are used.

In Figure 3a the predicted concentrations for all elements and all reference materials are plotted as a function of their certified concentrations. Predictions are largely oriented around the diagonal, especially for concentrations above 1%. In the range of 0.1–1% the network predicts higher values than certified. Below a concentration of 0.1% the deviations become larger. This is due to mostly low fluorescence intensities for concentrations below 0.1% and will be discussed later.

A similar plot for the density ρ can be seen in Figure 3b. The network performs well in the density range 0.5–3.5 g/cm^3^ with deviations below 30% yet fails to predict higher densities than 3.5 g/cm^3^, which is discussed in the next chapter.

In Figure 3c, the predicted surface positions *x*_0_ as a function of the certified surface position can be seen. Here, the alignment to the diagonal blue line is overall good, indicating that the network is able to predict the surface position well. A prominent outlier is the sample NIST 697 Bauxite represented by gray right triangular markers in the lower right. A detailed discussion is given in the following. The quantitative evaluation of the performance of the neural network is split into two parts, first the prediction of the surface *x*_0_ and the density ρ is evaluated and then the quantification of elemental concentrations.

## Density and Surface Quantification

The determination of sample densities is not an easy task, and established methods are not applicable to every sample. Thus, density determination with CMXRF would be highly beneficial. The certified densities for each reference material are calculated based on their weight and their volume with the assumption that they have a perfect cylindrical shape.

For all CRMs except the dense materials BAM M387 and Ferroetalon S25 the deviations of the predicted to the measured density are well below 30%. The training used samples with densities in the range of 0.5–3.5 g/cm^3^. To assess the model’s generalization capabilities, these two high-density metal samples were included, resulting in prediction failure. To improve performance, samples with higher densities must be simulated and included in the training.

Network predictions of the density show good precision, enabling exploratory use in determining the densities of unknown samples with CMXRF.

The surface position is not a fixed, certifiable value of the sample but depends on the lateral measurement position. The affirmed ground truth surface positions of the samples were determined using the software AbsCor, which performs FP quantification using an analytical fitting routine. For most depth-profiles the surface position was predicted within a range of ±10 μm of the certified position. Higher deviations can be found for inhomogeneous samples. This is to be expected since the network is solely trained on depth-profiles on homogeneous samples. Inhomogeneities lead to distorted depth-profiles rendering them unsuitable for this model. To overcome this, samples with inhomogeneities have to be included in the training.

Nonetheless, for NIST 697 Bauxite, which has typical depth-profiles without structures due to inhomogeneities, predictions are always too low and resulting deviations are high. To interpret this behavior, the package Captum^33^ was utilized to analyze which part of the depth-profile the network interprets as important. Notable for all depth-profiles is that the NN sets the fluorescence lines as an important feature. For the surface position signals of K and Ca are of highest importance for all CRMs where these elements are present. For NIST 697 Bauxite little K and Ca are present thus the network has to rely on other fluorescence lines, resulting in higher deviations. The shift to predict lower surface positions can also be explained this way. The network predicts the surface position using a specific distance to the maximum of the Ca/K K fluorescence profile. If no or little K/Ca is in the sample, the network will use fluorescence profiles of higher energy while keeping the same distance value for prediction. Due to the decrease of the size of the probing volume for higher energies, the real distance is overestimated resulting in much lower surface positions than certified. The same behavior can also be found in BR A4, BR C3 and BR E3, where K and Ca are also scarce. Plots of the extracted importance feature utilizing Captum version 0.7.0 on the depth-profiles of NIST 1577 Bovine Liver as a good performing example and NIST 697 Bauxite can be found in Figure S3.

Summarizing the prediction works well with higher deviations for inhomogeneous samples as well as samples outside the training data set. This is expected since NN show disadvantages in extrapolatory tasks.^34^ A plot showing the deviations for the density and surface position can be found in Figure S2.

## Concentration Quantification

**Figure 4:**
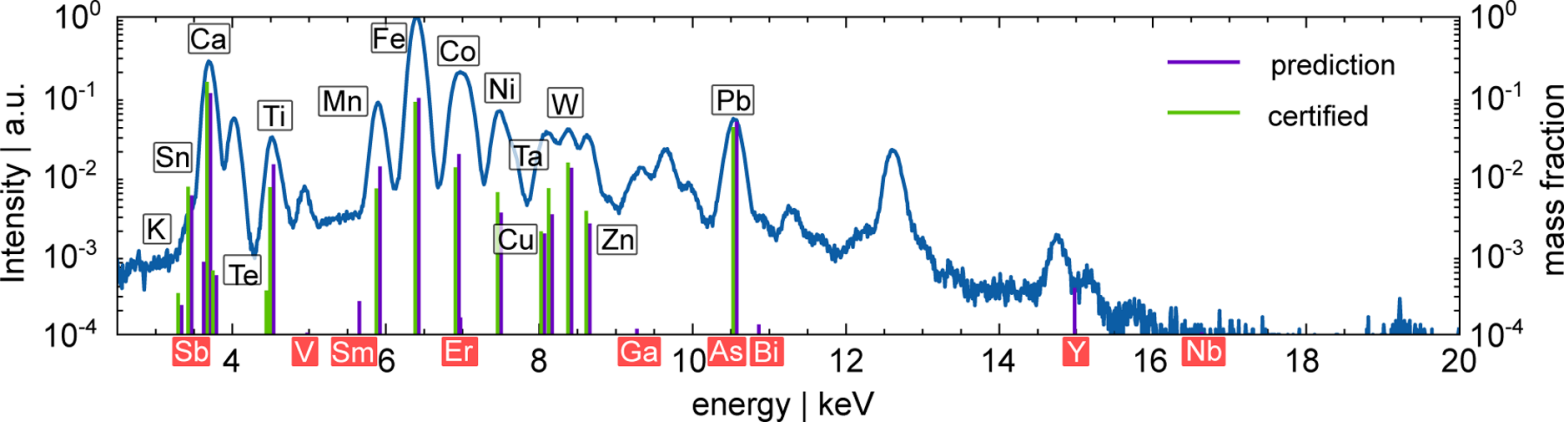
Plotted is a sum spectrum of one depth-profile of BR B2. The green vertical lines represent the certified concentrations and the purple lines the predicted concentrations. The network recognizes all measured element peaks.

In order to predict concentration values, the network needs to extract the fluorescence peaks. To validate if the network is detecting the correct fluorescence peaks, the elemental concentration predictions of elements are plotted in a spectrum at their corresponding fluorescence energy. Figure 4 shows the normalized sum spectrum of a BR B2 depth-profile, plotted in blue. Predicted elemental concentrations are shown as purple vertical lines at the specific fluorescence energies, alongside green vertical lines at the same positions representing the certified values. Elements corresponding to the fluorescence peaks are labeled near their respective peak positions. Labels for predicted elements which are not present are filled with red and positioned under the horizontal axis. The sample contains many fluorescence elements resulting in overlapping fluorescence lines. Even in such a complex spectrum the network is able to clearly detect the most intense fluorescence lines and predict their concentrations with low deviations. The predicted elements at low concentrations, e.g. Sb, Te, Er, Ga, As and Bi are at energy positions where peaks from other elements are present, thus indicating, that the network is not able to distinguish all fluorescence lines perfectly and rather predicts low concentrations than none. The predicted elements Y and Nb are at the edge of the setup’s sensitivity. Here, fluorescence intensities are generally low, and noise is prevailing, resulting in higher uncertainties from the NN. Also, the overestimated background by XMI-MSIM leads to higher deviation for elements with low concentrations. The concentration sum for nonpresent elements is below 0.5% for most CRMs.

Overall, the network is able to detect fluorescence peaks well and extract the relevant data from the spectra. Yet for low counts and overlapping peaks deviations are higher. It also tends to predict low concentrations instead of zero concentrations. Therefore, predicted low concentrations and concentrations at the edge of the setup sensitivity must be handled with care. Both issues may be addressed with additional training data, stricter prediction constraints, improved simulation of the background, or more advanced network architectures.

Analyzed samples consist mostly of elements to which CMXRF is not sensitive, with the dark matrix accounting for more than 65% of all samples and often exceeding 90%. While these elements cannot be predicted by the NN, they still influence absorption and scattering, altering the measured spectra and depth-profiles. Through these depth-profiles, the NN can learn to account for these influences and accurately predict concentrations for the detectable elements. For all CRMs the sum of the predicted concentrations is similar to the certified concentrations, thus indicating that the NN can interpret the influence of the dark matrix well. The test for the extrapolation capabilities of the NN on the alloy BAM M387 and steel Ferroetalon S25 again results in high deviations for those samples.

The results of the elemental quantification will be discussed using two aspects, first by analyzing the influence of the sample composition on the prediction and second by analyzing the influence of the analyzed element on the prediction.

## Influence of Sample Composition

To analyze the influence of the sample composition on the prediction, the predicted concentrations are plotted as a function of their certified concentrations for each reference material, similar to Figure 3a. A correlation of prediction accuracy and sample composition would show significant deviations for specific classes of material, e.g. glass or biological specimen with specific dark matrices. By analyzing all plots, the accuracy is independent of the sample composition.

The t-SNE analysis shows samples on the edge of the lower dimensional distribution, see e.g. BR A4. Yet the position in the distribution does not have a clear correlation with the quantification accuracy since the results for BR A4 have a good accuracy.

**Figure 5:**
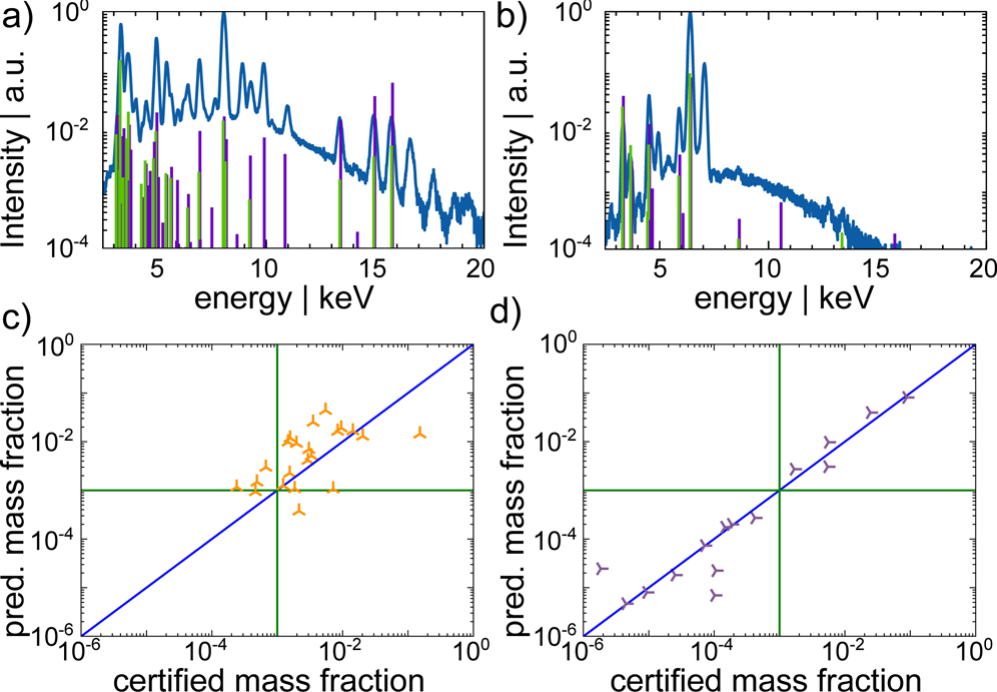
Plotted are normalized sum spectra of one depth-profile on the samples (a) BR F3 and (b) NIST 679 Brick. Predicted concentrations values as a function of the certified for all elements present in (c) BR F3 and (d) NIST 679 Brick are plotted. The blue line represents perfect agreement. If the fluorescence lines are separatable the model predicts accurately, if many elements with similar concentrations and overlapping fluorescence lines are present, the network tends to predict higher concentrations. The green lines mark concentrations of 0.1%.

The composition of the sample does have an influence on the prediction after all. If many elements are present and many fluorescence lines overlap, as can be seen exemplary for BR F3 in Figure 5a, the network cannot separate the fluorescence lines properly. In such a case, it seems the detected fluorescence intensity is assigned by the network to all involved elements without splitting it between them properly, leading to higher predicted concentrations for each concerned element. This can be seen in Figure 5c. Whereas, if the fluorescence lines are well distinguishable as can be seen exemplary for NIST 679 Brick in Figure 5b, the network predicts accurately even for elements with low concentrations. In Figure 5d the predictions are well aligned along the diagonal.

## Influence of Fluorescence Energy

**Figure 6:**
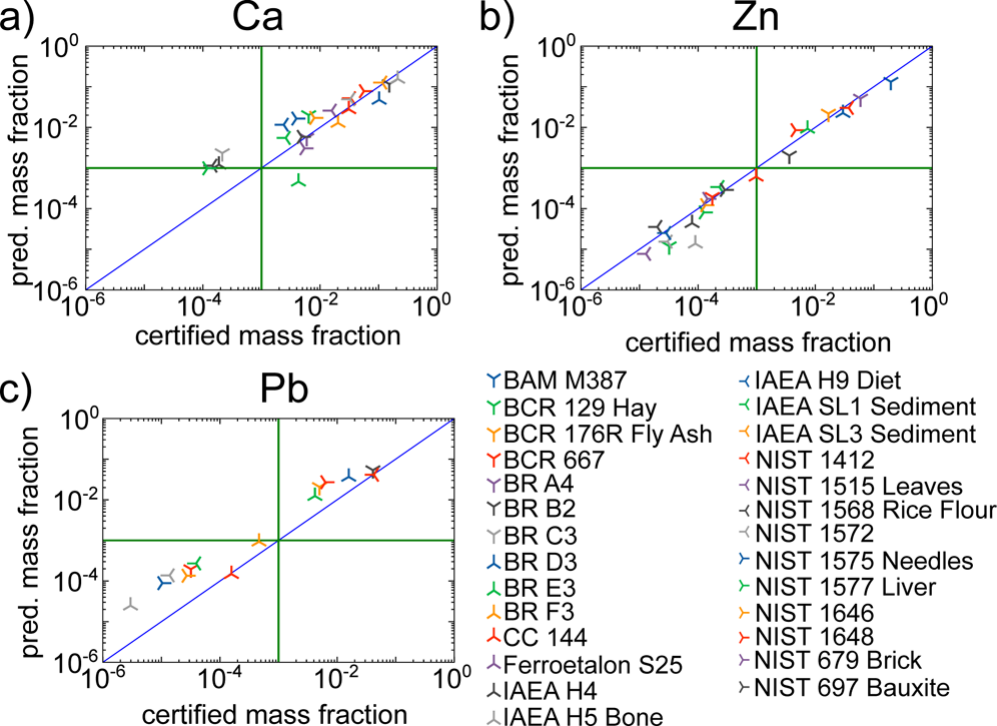
Plotted are the predicted concentrations as a function of the certified concentrations for the elements (a) Ca, (b) Zn and (c) Pb. The blue line indicates perfect agreement, the green lines a concentration of 0.1%.

To assess the influence of the fluorescence energy of each element on the prediction accuracy, the predicted concentrations for each sample are plotted against the certified concentrations for each element separately. In Figure 6 these plots are shown for Ca in Figure 6a, Zn in Figure 6b and Pb in Figure 6c. For Zn the predictions are accurate for the whole displayed concentration range. For Ca deviations to higher concentrations below concentrations of 1% are visible. These deviations are due to overlapping fluorescence lines in the lower energy range and the higher absorption in this energy range. Similar behavior can be seen for K, which is expected since K and Ca K fluorescence overlaps. The predictions for Pb are mostly higher than certified. One reason is that in all samples with higher deviations As is present, mostly in similar concentrations. Due to the proximity of the Pb L and As K fluorescence lines, distinguishing between the two is challenging. For lower concentrations the fluorescence yield for Pb is low, resulting in low fluorescence intensities and, therefore, higher expected deviations. Another reason could be the comparably wide distribution of L fluorescence lines over the energy. The NN architecture may not be suitable to create a connection between all fluorescence lines of one element if they are too far apart. This might be attributed to the convolutions’ receptive field.^35^ This could be improved by utilizing attention mechanisms^16^ or advanced architectures adapted for feature extraction like autoencoders.^18^

Elemental concentration quantification results are listed in the Table S5 alongside their certified concentrations and with quantification results from Förste et al.^23^ Overall, the quantification of the network shows good predictive accuracy with deviations below 30% for concentrations over 0.1%. For lower concentrations, the predicted concentrations are mostly in the correct order of magnitude. The NN predictions exhibit higher deviations than the classical analytical approach. This can be explained by deviations in the simulation and the restricted amount of training data. It can be improved by improving the simulation in order to enhance the background accuracy and by introducing a larger training data set.

## Unknown Samples

**Figure 7:**
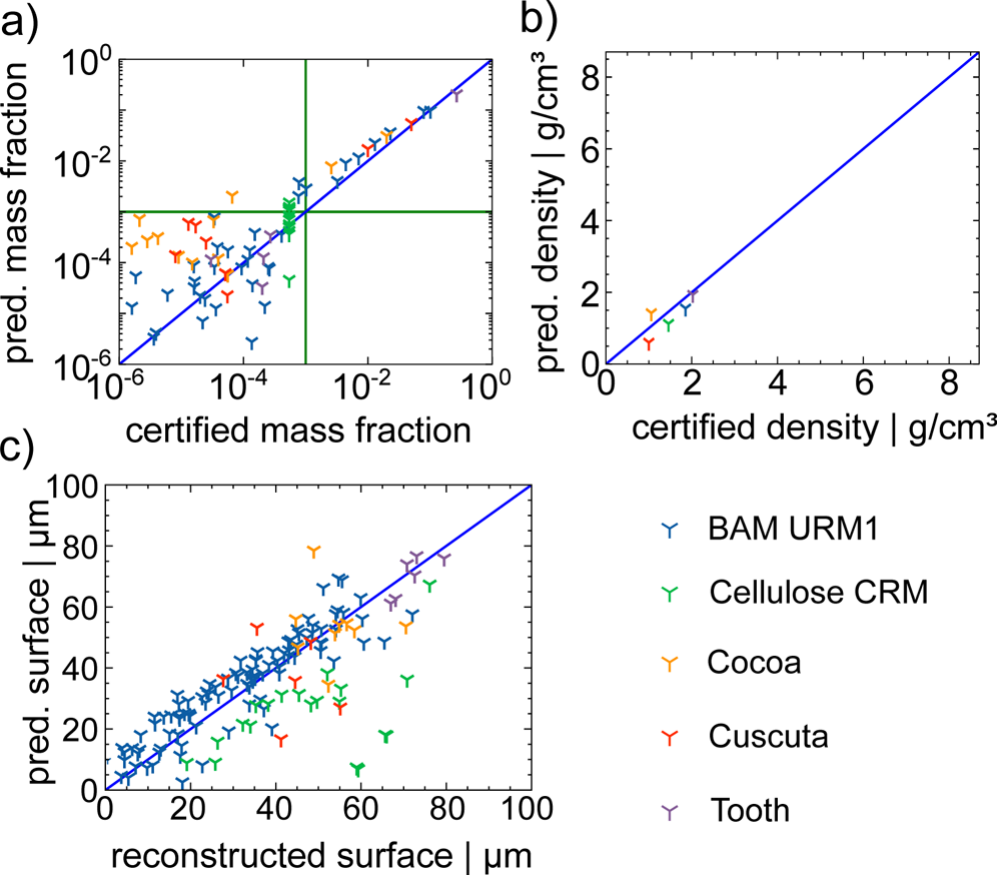
Plotted are the predicted values as a function of the certified for (a) concentrations, (b) surface position *x*_0_ and (c) density of all unreferenced materials, represented with different symbols. The blue line represents perfect agreement. The green lines mark concentrations of 0.1%.

Neural network quantification was applied to real-world samples, including BAM URM1, cellulose CRM, Cocoa, *C. reflexa*, and a cross section of a bovine tooth. Concentration values for comparison were sourced from previous publications.^9,21,22,36,37^ Quantification results are shown in Figure 7, exhibiting similar behavior to previously analyzed CRMs. Predicted densities, plotted in Figure 7b, show low deviations, as expected, since they fall within the range of 0.5–3.5 g/cm^3^. Surface predictions are presented in Figure 7c. Higher deviations are found for Cellulose CRM which can be explained by slight inhomogeneities in the sample resulting in distorted depth-profiles.

For the concentrations, which are plotted in Figure 7a, the accuracy is quite high for concentrations above 0.1% with higher deviations for lower concentrations.

This concludes that the training data set does not only reflect the CRMs but also the unknown biological samples. The NN can be used for the exploratory analysis of the density and elemental composition of unknown samples within a similar parameter space as the training data. This includes biological, glass and geological samples with a homogeneous distribution. For layered or inhomogeneous samples higher deviations will occur. The quantified concentrations can be found in Table S7.

## Conclusion

In this work, the implementation of a CNN for the quantification of CMXRF depth-profiles was introduced. Training was based solely on synthetic data, while evaluation was based on measured data. The procedure and quality of the simulated data was validated, and the simulations are in good agreement with measured samples with a deviation in the fluorescence lines well below 10%. An increased background in the simulation introduces higher deviations for low concentrated elements. The elemental composition of simulated samples was estimated on the analysis of elemental distributions in the reference material database GeoReM. It was shown that the elemental composition of the simulated samples reflect the parameter space of the measured materials analyzed in this work. The utilized NN was trained on 22,080 synthetic samples, including depth-profiles, with different setup parameters each. The performance of the NN was evaluated for the quantification of the surface position, the sample density and the elemental concentrations. Overall, 2152 depth-profiles on 27 CRMs were used for validation. The quantification with average 12 ms per depth-profile is fast, 100,000 times faster than the FP approach with about 20 s per element. The network can predict the density and surface position of a sample with high accuracy, with deviations below 30% for densities and ±10 μm for the surface position.

The accuracy of the prediction of elemental concentrations is generally high for concentrations above 0.1%, yet it is not as accurate as the FP approach discussed in Mantouvalou et al.^12^ where substantial human input is needed. Especially for samples with many overlapping fluorescence lines deviations of 1 order of magnitude are possible with the here presented approach, see e.g. Fe in BR A4 and BR F3. Quantification results should therefore be treated with adequate scrutiny. To improve accuracy the predicted values can be used as starting parameters for the classical FP quantification approach, minimizing human input and computation time, as fewer iterations will be necessary.

The accuracy of the concentration prediction depends on multiple influences which also influence FP quantification. The first influence is the sensitivity of the setup itself. At the edges of the sensitivity the predictions show higher deviations as compared to the regions with highest sensitivity. The occurrence of overlapping fluorescence lines in the measured spectra influences the accuracy. Elements with fluorescence lines in the energy range below 4 keV are most affected, since in this region many fluorescence lines are present. Overlapping fluorescence lines results in higher concentration predictions and the prediction of nonpresent elements. A neural network architecture for improved feature extractions, using e.g. autoencoders,^18^ could help improve the accuracy for elements with overlapping fluorescence lines. The concentration sum of predicted non present elements is generally below 0.5%. Another influence are inhomogeneities in the measured depth-profiles. The network was trained on purely depth-profiles on homogeneous samples. Therefore, any inhomogeneity leads to higher deviations.

Even though only a small fraction of the elements present in the samples are detectable with CMXRF, the sum of the predicted concentrations by the network is similar to the sum of the certified concentrations for the majority of the CRMs. This indicates that the NN understands the overall composition and the dark matrix of the samples. With further improvements of the NN, it could possibly be used to determine the dark matrix composition from CMXRF depth-profiles.

Overall, the network performs well, even with such a small training data set. This indicates that the training data set matches measured samples well. The performance of the network is expected to be improved by training on a larger data set. To improve the network’s generalization and robustness the training data set should be expanded by spectra which include multiple overlapping fluorescence lines and detector artifacts such as pile-ups. For further improvements the data set should contain a wider density range of the samples and including inhomogeneities. More training data and advanced automated machine learning,^38^ especially neural architecture search,^39^ could improve the model. To prevent the model from predicting small concentrations rather than no concentration, stronger noise during the training could be beneficial. Also, further restrictions on the output or adapted loss functions can help improve the performance of the NN.

In summary, the network allows fast quantification results without confocal setup characterization and long optimization routines. The possibility to reliably determine the density with CMXRF adds another feature to CMXRF investigations. Its easy accessibility, nondestructiveness and minor sample preparations makes it an ideal method for density determination. The surface prediction allows to perform surface topography investigations,^40^ and is the prerequisite for multilayer depth profiling quantification analysis. Currently, analytical 3D elemental reconstruction is only possible for multilayers and samples where the geometry and density is known. When expanding the surface prediction capabilities, NN might pave the way for a more generalized reconstruction possibility of truly heterogeneous objects.

In conclusion, the presented NN quantification opens a new way to efficiently analyze CMXRF data. While the accuracy is smaller than the commonly used analytical approach, the speed of analysis combined with minimal to no human input will further expand the applicability of the technique to various analytical fields in research and industry.

## Supporting Information Available

Table S1: times and quantity of measurements; Figure S1: t-SNE analysis of samples; Table S2: elemental distribution parameters; detailed MXRF-CMXRF transformation description; Table S3: setup parameters; Table S4: model architecture; Figure S2: Density and surface prediction deviations; Table S5: Quantification results and FP comparison; S6: Quantification results without FP comparison; Table S7: Quantification on unknown samples; Figure S3: Heat map for attribute importance for surface prediction (PDF)
