# Supplementary material for: Neural Networks for Quantifying Laboratory Confocal Micro X-ray Fluorescence Measurements
Source: Anal Chem. 2025 Mar 27;97(13):7177–85. doi: 10.1021/acs.analchem.4c06545 (PMC11983374; doi:10.1021/acs.analchem.4c06545)
Supplement: Supplementary file 1 — ac4c06545_si_001.pdf [file ac4c06545_si_001.pdf]

Supporting Information

for

Neural Networks for Quantifying Laboratory Confocal Micro X-Ray Fluorescence Measurements

Frank Förste[1], Leona Bauer[1], Yannick Wagener[1], Felix Hilgerdenaar[1], Felix Möller[2], Birgit Kanngießer[1,2], Ioanna Mantouvalou[2]

1 Institute for Optics and Atomic Physics, Technical University of Berlin, Berlin 10623, Germany

2 Helmholtz-Zentrum Berlin for Materials and Energy, Berlin 14109, Germany

Table of Contents

Page S2	Table S1. Listed are the number of measured and analyzed depth-profiles and measurement times utilized for every CRM.
Page S3	Figure S1. This plot shows the t-SNE dimension reduced representation of the elemental concentrations of all validation samples (red marker) and all 22080 simulated samples (blue marker). The validation samples lie within the simulated samples.
Page S4	Table S1. Listed are the log normal distribution parameter μ and σ and the occurrence rate for every analyzed element.
Page S5	Chapter: TRANSFORMATION OF MXRF TO CMXRF DATA
Page S6	Table S2. The values of the 6 setup parameters are drawn from a normal distribution with the given μ and σ. The surface position x0 and density ρ are drawn from a uniform distribution.
Page S6	Table S3. Listed is the architecture of the utilized neural model.
Page S7	Figure S1 Plotted are a) the relative deviations of the mean densities of the different CRMs and b) the absolute deviations of the surface position x_0 for each depth profile.
Page S7 – S10	Table S5. Mean quantification results from the Neural Network and the quantification routine based on the fundamental parameter.
Page S11	Table S6. Listed are quantified values of reference materials.
Page S12	Table S7. Listed are the certified and mean quantification results from the Neural Network and the deviation for the unknown samples.
Page S13	Figure S2. Plotted are in the upper plot the depth spectra maps (CMXRF spectra as a function of depth) and below the attributions or importance heat maps for the surface prediction derived from Captum for a) NIST 1577 Bovine Liver and b) NIST 697 Bauxite.

Table S4. Listed are the number of measured and analyzed depth-profiles and measurement times utilized for every CRM.

CRM	# depth-profiles	Measurement Times | s
BCR 129	100	20
BCR 176R	100	20
BCR 667	81	20
BR A4	4	20, 15, 30
BR B2	2	20
BR C3	2	20
BR D3	2	20
BR E3	2	20
BR F3	5	120, 20
CC 144	100	20
IAEA H4	100	20
IAEA H5	289	20
IAEA H9	100	15
IAEA SL1	100	15
IAEA SL3	100	15
BAM M387	144	10
S25	10	5
NIST 679	90	20
NIST 697	90	20
NIST 1412	80	20
NIST 1515	90	20
NIST 1568	100	18
NIST 1572	81	20
NIST 1575	100	20
NIST 1577	90	20
NIST 1646	90	20
NIST 1648	100	10
BAM URM1	100	15
Cellulose CRM	20	100
Cocoa	11	700
Cuscuta	6	200
Bovine Tooth	6	120

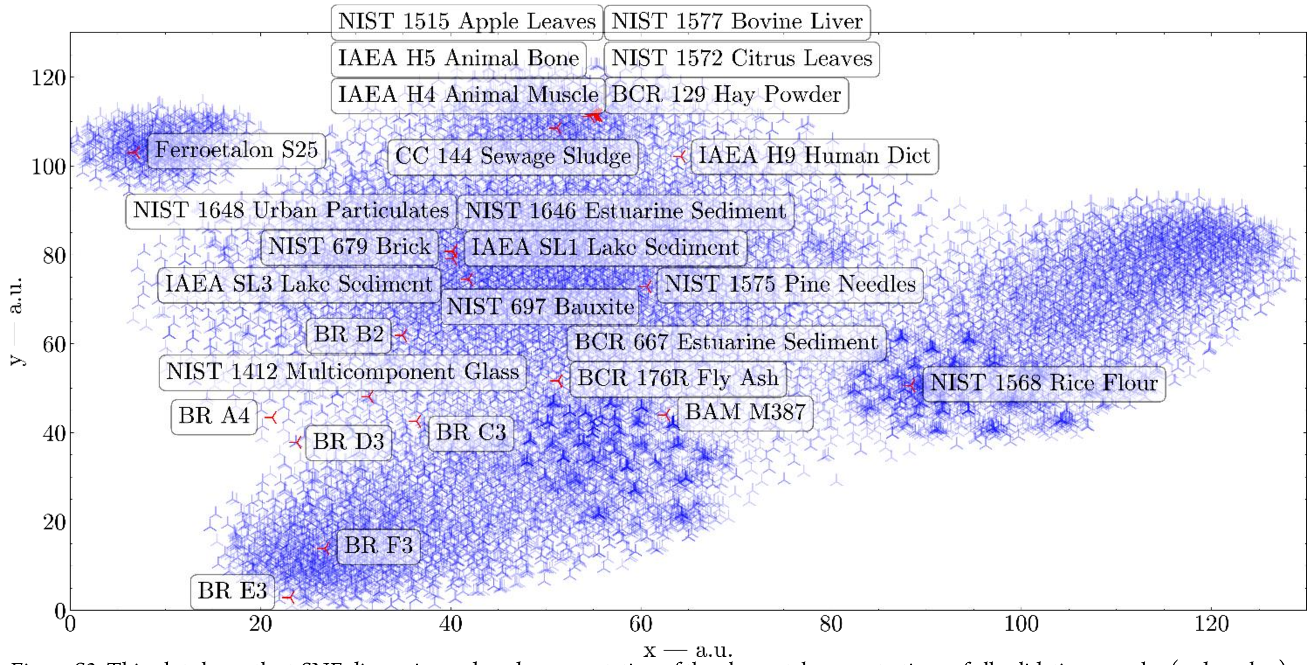

Figure S3. This plot shows the t-SNE dimension reduced representation of the elemental concentrations of all validation samples (red marker) and all 22080 simulated samples (blue marker). The validation samples lie within the simulated samples.

Table S5. Listed are the log normal distribution parameter μ and σ and the occurrence rate for every analyzed element.

Element	σ	μ	Occurrence Rate	E	σ	μ	O.R.	E	σ	μ	O.R.
H	4.68	3.47	0.46	C	0.15	1.06	0.51	N	2.7	2.64	0.18
O	-0.03	0.94	0.92	F	5.53	3.62	0.23	Na	3.23	2.94	0.65
Mg	2.59	2.75	0.68	Al	0.55	1.8	0.68	Si	-0.19	0.91	0.78
P	4.6	3.43	0.55	S	3.65	3.2	0.48	Cl	5.25	3.61	0.26
K	2.11	2.57	0.65	Ca	1	2.14	0.7	Sc	9.5	4.55	0.36
Ti	2.57	2.84	0.58	V	7.19	4.03	0.42	Cr	6.96	4.04	0.53
Mn	5.08	3.45	0.65	Fe	0.79	1.85	0.72	Co	8.07	4.32	0.48
Ni	6.76	4.06	0.5	Cu	6.83	4.06	0.52	Zn	6.49	3.89	0.53
Ga	8.05	4.26	0.29	Ge	10.85	4.81	0.21	As	7.96	4.37	0.43
Se	10.42	4.94	0.31	Br	8.83	4.56	0.2	Rb	6.62	3.99	0.39
Sr	4.64	3.5	0.43	Y	7.12	4.09	0.34	Zr	4.8	3.55	0.34
Nb	7.22	4.15	0.28	Mo	9.29	4.62	0.34	Ru	14.01	5.69	0.05
Rh	13.38	5.52	0.06	Pd	13.03	5.42	0.1	Ag	10.63	4.93	0.29
Cd	10.32	4.89	0.37	In	12.53	5.2	0.16	Sn	8.88	4.44	0.31
Sb	9.16	4.64	0.36	Te	12.14	5.21	0.15	I	9.62	4.76	0.14
Cs	8.88	4.54	0.34	Ba	4.35	3.33	0.43	La	7.19	4.07	0.38
Ce	6.24	3.86	0.37	Pr	8.22	4.36	0.29	Nd	7.75	4.14	0.33
Sm	8.98	4.46	0.34	Eu	9.38	4.68	0.34	Gd	8.95	4.44	0.29
Tb	10.3	4.8	0.31	Dy	8.98	4.46	0.3	Ho	9.74	4.72	0.28
Er	9.96	4.63	0.29	Tm	11.19	4.96	0.27	Yb	9.81	4.61	0.35
Lu	11.18	4.95	0.32	Hf	8.73	4.42	0.31	Ta	8.78	4.56	0.27
W	8.72	4.45	0.24	Re	17.26	6.14	0.06	Os	16.19	6.03	0.05
Ir	15.07	5.82	0.08	Pt	10.96	5.04	0.1	Au	13.42	5.47	0.18
Hg	11.54	5.15	0.27	Tl	9.53	4.72	0.23	Pb	6.59	3.95	0.51
Bi	9.57	4.71	0.23								

TRANSFORMATION OF MXRF TO CMXRF DATA

To convert an MXRF spectrum to a depth dependent CMXRF depth profile a procedure based on the fundamental parameter equation described in Förste et al. [1] was developed. The energy dependent transmission $T(E)$ and spot size $\sigma(E)$ of the second optic in the detection path have to be taken into account. The transmission of a polycapillary optic can be described by a Gumbel distribution [2]

$$T(E) = T_A \cdot e^{-e^{-z}-z+1} + T_0 \text{ with } z = \frac{E-T_M}{T_B} \tag{1}$$

with energy E, T_A the maximum transmission, T_M the energy of the maximum transmission, T_B the width of the transmission and T_0 the offset transmission. The spot size $\sigma(E)$ of the optic on the other hand can be approximated by a decaying exponential equation

$$\sigma(E) = \sigma^{MAX} e^{-\sigma^{ECP} \cdot E} + \sigma^{OFF} \tag{2}$$

with $\sigma^{MAX}$the hypothetical maximum spot size at E=0 keV, $\sigma^{\exp}$ the exponential decaying factor and σ^{OFF} the offset or minimal spot size. Due to the depth resolving nature of CMXRF, the absorption of the radiation in the sample must be considered. For a specific depth x_n the absorption can be described by the term

$$A(E, x_n) = e^{-\mu_{lin}(E)\cdot(x_n - x_0)} \tag{3}$$

with μ_{lin} as the linear absorption coefficient and x_0 as the relative surface position of the sample. The size of the probing volume leads to an enhanced intensity at the measurement position which can be described by the term

$$B(E) = e^{\frac{(\mu_{lin}(E)\cdot\sigma(E))^2}{2}} \tag{4}$$

The size of the probing volume also introduces a term describing the convolution of the volume with step-like layer boundaries, e.g. when the probing volume enters the sample. It can be calculated at depth position x_n as

$$\frac{1}{2}\left[erf\left(\frac{x_0 + D + \mu_{lin}(E)\sigma^2(E) - x_n}{\sqrt{2}\sigma(E)}\right) - erf\left(\frac{x_0 + \mu_{lin}(E)\sigma^2(E) - x_n}{\sqrt{2}\sigma(E)}\right)\right] \tag{5}$$

with D the thickness of the sample. For infinitely thick samples as considered in this study, the equation (5) can be simplified to

$$C(E, x_n) = \frac{1}{2}\left[1 - erf\left(\frac{x_0 + \mu_{lin}(E)\sigma^2(E) - x_n}{\sqrt{2}\sigma(E)}\right)\right] \tag{6}$$

The transformation from an MXRF spectrum to a CMXRF depth-profile can thus be conducted by calculating the following equation for each depth x_n relative to the surface relative position x_0

$$\Phi(E, x_n) = \Phi_0(E) A(E, x_n) \cdot B(E) \cdot C(E, x_n) \cdot T(E) \tag{7}$$

with $\Phi(E, x_n)$ fluorescence intensity at energy E and position x_n and $\Phi_0(E)$ the MXRF spectrum. The transformation can thus be performed using the 6 setup parameters T_A, T_B, T_M, σ^{MAX}, $\sigma^{\exp}$ and σ^{OFF} and the surface position x_0.

Table S6. The values of the 6 setup parameters are drawn from a normal distribution with the given μ and σ. The surface position x0 and density ρ are drawn from a uniform distribution.

T_A	0	0.003
T_B	0	0.053
T_M	0	0.05
σ^{MAX}	0	1.67
σ^{EXP}	0	0.003
σ^{OFF}	0	0.13
x_0	low=0	high=300
ρ	low=0.5	high=3.5

Table S7. Listed is the architecture of the utilized neural model.

CNN	Conv2D(in_channels=1, out_channels=15, kernel_size=(3,4))<br>Mish()<br>MaxPool2D(kernel_size=(3,4))<br>Conv2D(in_channels=15, out_channels=15, kernel_size=(2,2))<br>Mish()<br>MaxPool2D(kernel_size=(2,2)
MLP	Linear(in_features=10850, out_features=1200)<br>Mish()<br>Dropout(0.2)<br>Linear(in_features=1200, out_features=600)<br>Mish()<br>Linear(in_features=600, out_features=200)<br>Mish()<br>Linear(in_features=200, out_features=55)

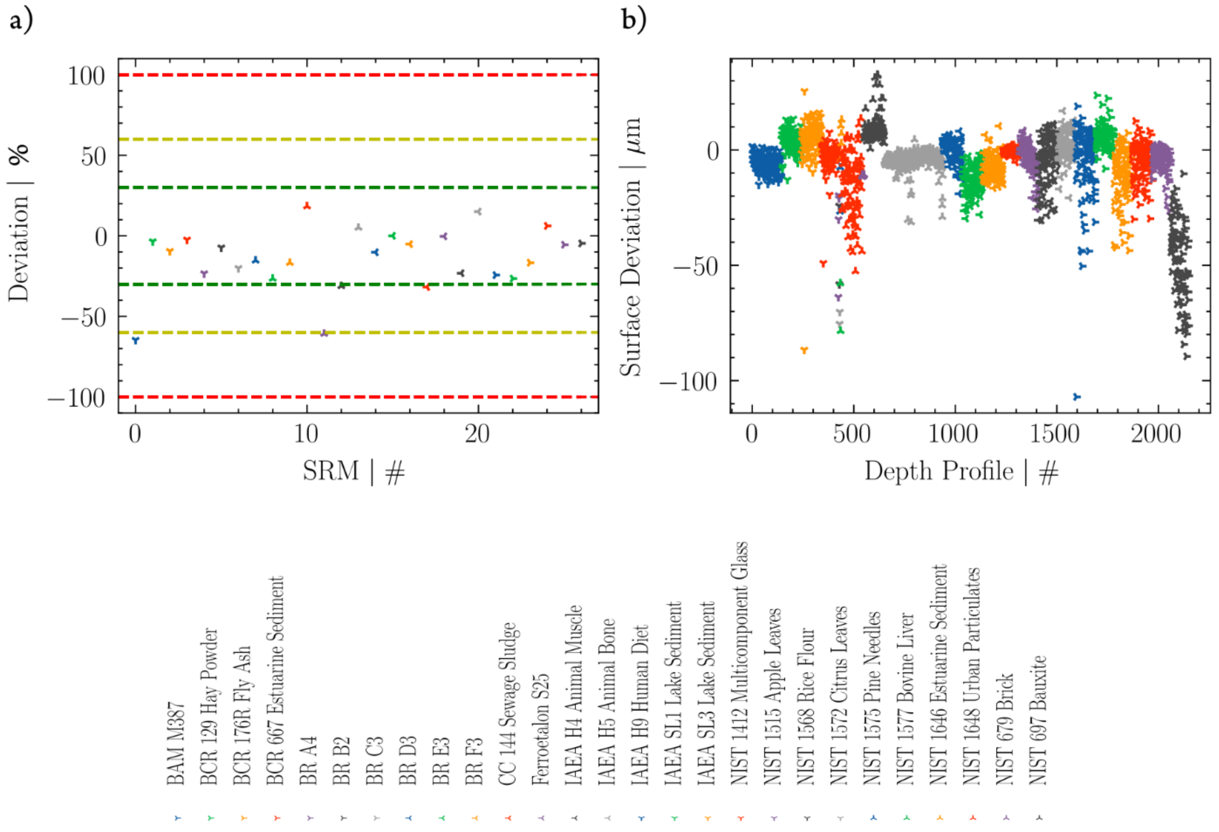

Figure S4 Plotted are a) the relative deviations of the mean densities of the different CRMs and b) the absolute deviations of the surface position x_0 for each depth profile.

Table S5. Mean quantification results from the Neural Network and the quantification routine based on the fundamental parameter.

Element	Cert conc | %	FP conc | %	Deviation | %	NN conc | %	SD %	Deviation | %
BR A4						
K	1.7931	—	—	1.6	0.8	-14.8
Ca	0.5932	0.5784 ± 0.0105	-2.5	0.23	0.17	-61.1
Ba	1.79	1.62 ± 0.05	-9.4	2.4	2.2	32.1
Ti	2.34	3.03 ± 0.06	29.6	1.7	0.7	-29.5
Cr	0.103	—	—	0.019	0.019	-81.9
Mn	15.7	14.9 ± 0.2	-5.2	11.9	2.7	-24.6
Fe	0.811	0.829 ± 0.015	2.2	1.99	1.07	145.2
Zn	5.95	5.67 ± 0.13	-4.6	6.1	1.5	2.4
Ge	0.056	0.072 ± 0.003	28.9	0.02	0.03	-65.1
Rb	0.037	0.115 ± 0.003	214.3	0.00091	0.00104	-97.5
Sr	0.6	—	—	2.9	1.7	385.6

BR B2						
K	0.032	—	—	0.008	0.006	-77.2
Sn	0.72	0.77 ± 0.03	5.7	0.6	0.9	-14.3
Ca	15.2	15.8 ± 0.2	3.7	17.2	5.8	13.1
Ti	0.719	0.790 ± 0.016	9.9	0.65	0.19	-10.2
Mn	0.689	0.852 ± 0.012	23.5	0.6	0.4	-12.1
Fe	8.53	8.67 ± 0.14	1.6	10.8	4.8	26.2
Co	1.27	1.37 ± 0.03	7.6	0.6	0.4	-51.9
Ni	0.62	0.61 ± 0.02	-1.5	2.6	0.5	322.3
Cu	0.200	0.184 ± 0.004	-7.7	0.27	0.08	35.0
Ta	0.70	0.76 ± 0.03	9.1	0.85	1.12	22.2
W	1.47	1.30 ± 0.03	-11.6	1.9	0.6	30.5
Zn	0.362	0.367 ± 0.008	1.5	0.6	0.3	62.8
Pb	4.08E-02	—	—	2.5	0.4	-39.3
BR C3						
K	5.728	5.463 ± 0.109	-4.6	3.78	0.07	-33.9
Ba	0.896	0.870 ± 0.017	-2.8	0.8	0.3	-7.1
Ti	0.060	0.093 ± 0.004	54.5	0.040	0.005	-33.5
Pr	0.171	0.188 ± 0.007	9.8	0.024	0.009	-86.0
Nd	0.345	0.372 ± 0.005	7.7	0.6	0.4	72.7
Mn	0.364	0.392 ± 0.006	7.5	0.38	0.07	3.5
Fe	3.78	3.68 ± 0.06	-2.7	4.35	0.12	15.3
Ni	0.2279	0.2204 ± 0.0106	-3.3	0.16	0.05	-30.2
W	0.714	0.670 ± 0.012	-6.2	0.4	0.1	-47.5
Ge	0.187	0.256 ± 0.012	36.6	0.04	0.03	-79.5
As	0.591	0.725 ± 0.013	22.7	0.63	0.06	6.3
Bi	0.449	0.478 ± 0.011	6.5	0.29	0.16	-34.7
Ca	2.14E-02	—	—	0.05	0.04	119.6
BR D3						
K	7.47E-02	—	—	0.02	0.02	-67.7
Ca	10.2	11.1 ± 0.7	8.8	9.2	4.4	-9.8
La	0.75	0.84 ± 0.02	12.1	1.1	0.3	47.2
Ce	0.717	0.697 ± 0.019	-2.8	3.2	2.2	342.1
V	0.48	0.54 ± 0.02	12.8	0.7	0.2	38.2
Fe	0.41	0.46 ± 0.02	11.6	2.2	0.9	451.3
W	0.254	0.264 ± 0.008	3.9	0.19	0.03	-27.2
Zn	2.97	2.97 ± 0.06	-0.1	1.9	0.5	-36.0
Ga	0.342	0.364 ± 0.008	6.4	0.26	0.01	-23.1
Ge	0.285	0.298 ± 0.007	4.7	0.05	0.05	-81.0
As	1.41	1.61 ± 0.04	14.6	3.2	2.4	129.5
Sr	0.11	—	—	0.044	0.003	-59.6

BR E3						
K	0.789	0.777 ± 0.019	-1.5	2.07	1.14	162.1
Ba	4.12	4.71 ± 0.11	14.3	7.11	1.18	72.6
La	0.341	0.298 ± 0.012	-12.7	0.6	0.4	74.9
Cr	0.383	0.465 ± 0.009	21.3	0.2	0.2	-38.3
Mn	5.03	5.58 ± 0.09	10.8	2.9	1.5	-41.6
Co	0.5820	0.6270 ± 0.0112	7.7	0.8	0.6	42.5
Ni	1.45	1.40 ± 0.03	-3.9	1.1	0.2	-27.7
Cu	0.655	0.694 ± 0.018	5.9	2.1	0.9	226.2
Zn	0.74	0.84 ± 0.02	13.6	0.4	0.1	-46.7
As	0.333	0.567 ± 0.012	70	0.4	0.2	12.6
Sr	0.262	0.665 ± 0.014	153.8	0.8	0.7	202.4
BR F3						
K	15.2	13.5 ± 0.4	-11	9.6	4.0	-37.0
Ca	2.03	2.08 ± 0.05	2.5	2.4	1.2	18.8
V	0.95	1.34 ± 0.02	40.7	1.3	0.4	37.5
Sm	0.155	0.191 ± 0.004	22.8	0.2	0.1	49.0
Fe	0.049	0.078 ± 0.002	58.8	0.4	0.4	671.9
Co	0.197	0.217 ± 0.004	10.1	0.3	0.2	72.2
Cu	1.44	1.45 ± 0.03	0.8	0.8	0.5	-41.5
Ta	0.29	0.40 ± 0.01	37.3	1.3	0.7	341.3
Ga	0.0670	0.0712 ± 0.0018	6.2	0.18	0.19	172.5
BCR 129 Hay Powder						
K	3.38	2.81 ± 0.03	-16.8	6.7	1.9	98.1
Ca	0.64	0.70 ± 0.04	8.7	2.0	0.7	217.9
Mn	0.0072	0.0138 ± 0.0006	92	0.02	0.03	185.6
BCR 176R Fly Ash						
Mn	0.073	0.054 ± 0.002	-26.6	0.19	0.16	158.2
Fe	1.310	0.516 ± 0.015	-60.6	1.47	1.08	12.0
Ni	0.0117	0.0137 ± 0.0009	16.7	0.0005	0.0013	-96.1
Cu	0.105	0.141 ± 0.005	34.4	0.07	0.08	-32.9
Zn	1.68	1.44 ± 0.04	-14.5	2.1	0.9	24.9
Pb	0.500	0.490 ± 0.014	-2	0.6	0.6	28.9
BCR 667 Estuarine Sediment						
Cr	0.0178	0.0117 ± 0.0005	-34	0.010	0.012	-43.6
Mn	0.0920	0.0786 ± 0.0015	-14.5	0.15	0.09	61.5
Fe	4.5	4.6 ± 0.1	2.1	5.1	1.6	13.8
Ni	0.0128	0.0148 ± 0.0006	16	0.0085	0.0117	-33.7
Cu	0.0060	0.0152 ± 0.0004	154	0.0006	0.0012	-90.5

CC 144 Sewage Sludge						
K	0.290	0.223 ± 0.006	-23	0.30	0.13	3.3
Ca	3.10	2.82 ± 0.04	-9	3.75	1.14	21.1
Ti	0.150	0.176 ± 0.003	17.5	0.163	0.102	8.4
Cr	0.0168	0.0234 ± 0.0012	39.6	0.02	0.03	21.3
Mn	0.0352	0.0248 ± 0.0009	-29.6	0.04	0.03	8.4
Fe	3.29	2.45 ± 0.04	-25.7	3.1	1.1	-6.2
Ni	0.0091	0.0155 ± 0.0007	70.8	0.004	0.008	-52.5
Cu	0.0348	0.0330 ± 0.0006	-5.3	0.03	0.05	-19.6
NIST 697 Bauxite						
Cr	0.068	0.098 ± 0.004	43.1	0.12	0.14	74.1
Mn	0.32	0.42 ± 0.02	33.4	0.21	0.12	-32.6
Fe	14,0	16.9 ± 0.4	20.9	9.7	2.9	-30.7
NIST 1577 Bovine Liver						
Fe	0.0268	0.0418 ± 0.0006	55.9	0.08	0.06	181.1
Cu	0.0193	0.0223 ± 0.0009	15.4	0.03	0.03	65.9
NIST 1646 Estuarine Sediment						
Mn	0.038	0.074 ± 0.003	96	0.05	0.04	20.4
Fe	3.50	4.02 ± 0.12	14.9	4.2	1.6	19.6

Table S6. Listed are quantified values of reference materials.

Element	Cert Conc | %	NN conc | %	Deviation | %
BAM M387			
Mn	0.08	0.04	-54.2
Fe	0.06	0.0	32.3
Ni	5.02	3.52	-29.9
Cu	75.2	19.5	-74.1
Zn	19.6	12.6	-35.6
Ferroetalon S25			
Ti	0.46	0.51	11.8
Cr	15.6	3.1	-80.1
Mn	1.90	0.13	-93.3
Fe	64.7	11.7	-81.9
Ni	13.8	6.6	-51.9
Nb	0.07	0.01	-86.6
IAEA H4 Animal Muscle			
K	0.4	4.9	1135.1
Ca	0.02	0.13	575.8
IAEA H5 Animal Bone			
Ca	21.2	16.4	-22.5
IAEA H9 Human Diet			
K	0.83	2.52	203.6
Ca	0.23	1.16	404.1
IAEA SL1 Lake Sediment			
K	1.5	2.5	66.6
Ca	0.25	0.55	120.0
Ti	0.52	0.88	69.3
Mn	0.35	0.51	46.0
Fe	6.74	7.00	3.9
Zn	0.02	0.03	52.4
IAEA SL3 Lake Sediment			
K	0.87	1.43	63.7
Ca	11.1	12.7	14.1
Ti	0.26	0.33	27.2
Sr	0.47	0.14	-69.6
NIST 1412 Multicomponent Glass			
K	3.44	3.55	3.3
Ca	3.24	5.22	61.4
Zn	3.6	3.03	-15.9
Sr	3.85	6.44	67.3
Cd	3.83	0.62	-84.0
Ba	4.18	4.36	4.3
Pb	4.08	4.12	1.0
NIST 1515 Apple Leaves			
K	1.61	2.75	71.0
Ca	1.52	2.58	69.5
NIST 1568 Rice Flour			
K	0.11	0.86	666.3
Ca	0.01	0.11	716.5
NIST 1572 Citrus Leaves			
K	1.82	3.06	67.9
Ca	3.15	4.91	56.0
NIST 1575 Pine Needles			
K	0.37	1.87	404.8
Ca	0.41	1.65	302.3
Mn	0.07	0.11	57.7
NIST 1648 Urban Particulates			
K	1.06	1.77	67.2
Ca	5.84	7.79	33.4
Ti	0.40	0.89	120.8
Mn	0.08	0.20	154.4
Fe	3.92	4.84	23.5
Cu	0.06	0.16	157.7
Zn	0.48	0.86	78.1
As	0.01	0.03	172.9
Pb	0.66	2.7	312.2

Table S7. Listed are the certified and mean quantification results from the Neural Network and the deviation for the unknown samples.

Element	Cert conc | %	NN conc | %	Deviation | %
BAM URM1			
K	2.3	3.55	54.4
Ca	10.2	9.44	-7.0
Ti	0.71	1.14	60.7
V	0.012	0.011	-5.3
Mn	0.102	0.279	173.8
Fe	7.89	9.37	18.8
Cu	0.077	0.198	158.0
Zn	1.3	2.19	68.8
Sr	0.078	0.371	377.2
Sn	0.324	0.392	21.1
Ba	0.441	0.882	100.0
Cellulose SRM			
K	0.054	0.083	52.8
Ca	0.054	0.148	171.7
Cr	0.054	0.121	122.3
Mn	0.054	0.041	-24.9
Co	0.054	0.05	-8.7
Ni	0.054	0.079	45.8
Cu	0.054	0.068	24.7
Zn	0.054	0.035	-35.4
Ga	0.054	0.05	-8.4
Sr	0.054	0.048	-11.2
Cd	0.054	0.11	102.6
In	0.054	0.068	25.3
Ba	0.054	0.102	88.2
Bi	0.054	0.115	111.0
Cocoa			
K	2	3.01	50.3
Ca	0.258	0.765	196.8
Cuscuta			
K	5	5.39	7.9
Ca	1	1.67	66.5
Zahn			
Ca	26.8	19.9	-25.7
Zn	0.02	0.003	-83.8
Sr	0.027	0.034	24.0
Ba	0.021	0.012	-41.1

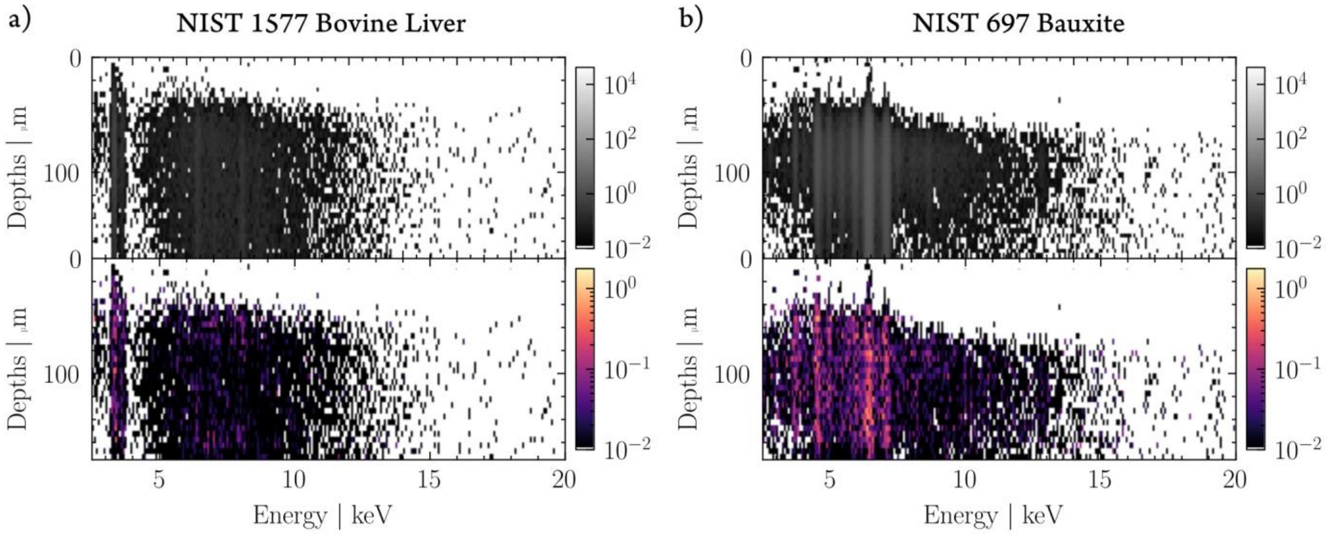

Figure S5. Plotted are in the upper plot the depth spectra maps (CMXRF spectra as a function of depth) and below the attributions or importance heat maps for the surface prediction derived from Captum for a) NIST 1577 Bovine Liver and b) NIST 697 Bauxite.
